# Motivational interviewing for the management of child and adolescent obesity: a systematic literature review

**DOI:** 10.3399/BJGPO.2022.0145

**Published:** 2023-12-13

**Authors:** Romain Lutaud, Eva Mitilian, Jenny Forte, Gaetan Gentile, Rachel Reynaud, Camille Truffet, Thibault Bellanger

**Affiliations:** 1 Département Universitaire de Médecine Générale, Faculté des Sciences Médicales et Paramédicales, Aix-Marseille Université, France; 2 CNRS, EFS, ADES, Aix-Marseille Université, Marseille, France; 3 Aix Marseille Université, Institut des Neurosciences des Systèmes, INSERM, France; 4 Aix Marseille Université, Faculté des Sciences Médicales et Paramédicales, Service de Pediatrie Multidisciplinaire CHU Timone Enfants, APHM, France

**Keywords:** motivational interviewing, overweight, obesity, child, adolescent, systematic review, primary healthcare, general practice

## Abstract

**Background:**

Among children or adolescents with obesity, 40–70.5% will remain obese as adults according to their paediatric body mass index (BMI). The recommended management involves changes in their nutritional habits (diet, physical activity, and sedentary lifestyle). Motivational interviewing (MI), a patient-centred consultation, has proven its worth in many fields where acting on behaviours is essential.

**Aim:**

To investigate the use and outcomes of MI in the management of children and adolescents who are overweight and obese.

**Design & setting:**

A systematic review evaluated MI in the management of children and adolescents who are overweight and obese.

**Method:**

PubMed, Web of Science, Cochrane Library, and CISMeF were searched between January 2022 and March 2022 for following terms: ‘motivational interviewing’, ‘overweight or obesity’, ‘children or adolescent’ to identify randomised controlled trials (RCTs). Inclusion criteria were interventions involving MI in children or adolescents who were commonly (polygenically) overweight or obese. Exclusion criteria were: studies before 1991; and articles not written in English or French. The first stage of the selection process was carried out by reading the titles and abstracts. A second stage was carried out by reading the complete studies. A secondary inclusion of articles was carried out following the reading of bibliographic references, mainly from systematic reviews and meta-analyses. The data were summarised in synthetic tables based on the Population, Intervention, Comparison, Outcomes, and Study (PICOS) tool.

**Results:**

From 444 articles the review identified 26 RCTs. Statistically significant results were found for all criteria (anthropometric and behavourial) in both children and adolescents. Quality of life and depression scores were also improved. Parental presence in the interview appeared to be essential for children, whereas for adolescents, the supportive involvement of parents outside of the interviews seemed more appropriate. The frequency and duration of the interventions played a major role in obtaining results, as did the number of people involved, and the diversity of the places where they are taken care of.

**Conclusion:**

MI seems promising for children and adolescents with overweight or obesity, within the framework of a comprehensive, multiprofessional, family management, carried out over a long period with regular consultations.

## How this fits in

MI, a patient-centred consultation, has proven its worth in many fields where acting on behaviours is essential. MI has shown promising results for the management of adult obesity. This review of the literature showed that MI is also a useful tool for the management of overweight and obesity in children and adolescents by GPs. Effects were found on behaviour change and anthropometric values.

## Introduction

The World Health Organization (WHO) described obesity in 1997 as an *‘international epidemic’*. In 2016, it counted 340 million children with obesity aged 5–19 years in the world.^1^

Preventing and managing childhood obesity is a complicated but crucial challenge. It is known that among children or adolescents with obesity, 40–70.5% will remain obese as adults, according to their paediatric BMI,^2^ and they will have an increased risk of secondary complications and a higher mortality rate.^3^ For children and teens, BMI is age and sex-specific and is often referred to as BMI-for-age. Overweight is defined by a BMI-for-age between the 85th percentile and the 95th percentile, while obesity is defined by a BMI-for-age above the 95th percentile

It is known that the causes (and therefore the therapeutic axes) of overweight or common (polygenic) obesity are mainly behavioural.^4^ MI is designed to enhance intrinsic motivation and promote confidence in a person’s ability to make behaviour changes.

MI is a patient-centred collaborative consultation technique,^5^ based on empathy and non-judgement. Its aim is to encourage the patient’s motivation to change, by leading them to reflect on their ambivalences, leaving them free to make their own decisions.^6^ A central tool is reflective listening, which aims at validating the understanding and which contribute to the trust between the two protagonists.^7,8^

In MI, the practitioner should always be careful to avoid resistance, which arises from opposition between the patient and the practitioner when the latter seeks to argue for change. Instead, the practitioner should promote autonomy and ‘self-efficacy’ (that is, the patient’s self-confidence in being able to undertake the actions necessary for change).^9^

The beneficial effects of MI have been proven on different addictions: first alcohol,^5^ then tobacco, cannabis, opioids.^6^ It permits a decrease in risk behaviours (for example, needle-sharing) but also the control of somatic pathologies such as diabetes or asthma (greater weight loss, more physical exercise, and a healthier diet).^10–12^

Several studies have focused on the management of obesity in adults with MI, which showed promising results. Although the findings were not obvious in terms of improvement in anthropometric criteria, there were notable effects on behavioural change.^13–15^

Among children and adolescents, various MI studies have also reported positive results concerning addiction problems (for example, tobacco, alcohol), risk behaviours (for example, use of seat belts or condoms), the management of HIV infection, and the prevention of caries by using MI during dental appointments.^16–18^

The objective of this systematic review was to investigate the use and outcomes of MI in the management of children and adolescents with overweight or obesity.

## Method

### Research protocol

To conduct this exploratory systematic literature review, the following bibliographic databases were searched: PubMed, Cochrane Library, Web of Science, and CISMeF. MeSH descriptors were used, associated with Boolean operators to create the following search equation: ‘motivational interviewing’ AND ‘overweight OR obesity’ AND ‘children OR adolescent’.

### Article selection

The first stage of selection was carried out by reading the titles and abstracts. A second stage was carried out by reading the complete studies. A secondary inclusion of articles was carried out following the reading of bibliographic references, mainly from systematic reviews and meta-analyses. Randomised controlled trials were selected, the first of which was published in 1991 (Table 1). The motivational interventions consisted of the one devised, described, and applied by Miller and Rollnick.^6^

**Table 1.** Inclusion and exclusion criteria

Inclusion criteria
Intervention involving motivational interviewing
Involving children or adolescents
Commonly overweight or obese (BMI-for-age >85^th^ percentile)
**Exclusion criteria**
Article published before 1991
Not written in French or English
Not available in full and not free of charge
Not a randomised controlled trial (RCT)
Involving children aged <4 years or >19 years
Not all children were overweight or obese
Concerning secondary or genetic obesity

### Data collection and writing

The methodological research and writing work was based on the Preferred Reporting Items for Systematic Reviews and Meta-Analysis (PRISMA).^19^ The data considered notable and homogeneous from the articles included in this review were summarised in synthetic tables based on the PICOS tool.^20^ Table 2 shows the grid used for data extraction.

**Table 2.** Data extraction

The data from the selected articles were extracted according to the following grid:
Study title First author and year of publication Country Study duration Number of participants included in the study Age of participants Presence or absence of parents Possible special feature of the study population Number of randomisation arms or analysis: intervention (E), intervention bis (E‘), control group (C) Description of intervention, duration, frequency, and so on Primary (Ir) and secondary (IIr) outcomes Main results

The main outcomes were selected and then divided in three categories of criteria.

First, anthropometric and clinical criteria, which included the following: BMI, abdominal circumference, abdominal circumference to height ratio, percentage of fat or lean mass, blood pressure (BP), VO_2_max, biological lipid exploration, glycaemia, insulin resistance.

Second, behavioural criteria: dietary (sugar, fat, fruits, vegetables), physical (sports activity), sedentary (screen time), motivation to change or adherence to follow-up.

Third, psychosocial criteria: feeling of personal effectiveness, quality of life, depression.

## Results

### Selected studies

**Figure 1.**
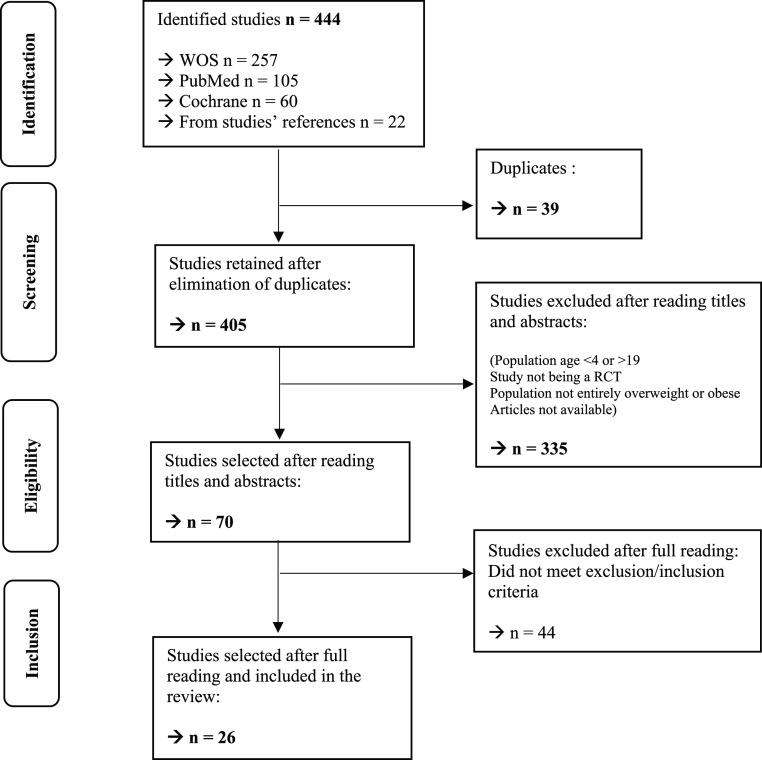
Flowchart. WOS = Web of Science; RCT = randomised controlled trial

The flowchart shows the different selection stages (Figure 1).

A total of 444 articles were identified and 26 were selected for inclusion in the final review after being read in full and meeting the inclusion and exclusion criteria.^21^ ^22–41^The reasons for exclusion were mainly the following: population aged <4 years or aged >19 years; study not being an RCT; study population not entirely overweight or obese; and articles not available. The abstracts of the selected articles and their main findings are available in Supplementary Table S1; this table provides a convenient and comprehensive overview of the literature reviewed, allowing readers to quickly and easily access the key information contained in each article.

### Motivational intervention procedures

#### Type of intervention

Thirteen studies compared MI with a control group. Ten studies compared the effectiveness of an existing programme between the MI and non-MI groups. Two studies compared the effects of MI between an adolescent–parent group, an adolescent-only group, and a control group without MI. Another study compared the presence or absence of parents without any control group.

The control groups were heterogeneous, ranging from having the same number of sessions as the MI group to no consultation. The number of visits in the control group was mostly lower than in the intervention group. These consultations corresponded to a classic follow-up with advice and guidelines, in a traditional directive interview style, according to the updated French national recommendations. Sometimes these consultations also involved provision of advice brochures to the patients.

#### Frequency and duration of intervention

The following three frequencies of intervention were reported:

- fewer than one session per month: from one every 6 weeks to one every 3 months;
- about one session per month: the average;
- more than one session per month: from two to four per month.

The duration of each session varied between 20 minutes and 60 minutes (average of 30 minutes). The duration of the studies ranged from 2 weeks–2 years (median of 6 months).

#### Locations and practitioners

The RCTs took place in 12 different countries (Supplementary Table S2). Nineteen studies were held in health centres (city paediatricians’ offices, GPs’ offices, paediatric clinics, hospital paediatric departments). Six studies were conducted in school settings for both recruitment and intervention. One study recruited African-American adolescent girls from churches.

Practitioners were most often physicians (paediatricians, psychiatrists) but also psychologists, nurses, dieticians, or professionals in sports activities.

#### Presence and role of the parents

Young children were accompanied to medical consultations by their parents (Table 3). Most of the children or adolescents were girls and most of the parents involved were mothers. This triad configuration makes it possible to value the changes in behaviour, and lead to a more lasting and non-stigmatising change. Three studies specifically examined the comparison of parent and non-parent MI practice. While one study found greater effectiveness in the parent–teen group,^42^ the other two found either no difference,^43^ or better results in the adolescent group alone.^44^

#### Training and fidelity control

The training of MI practitioners was reported in 23 out of 26 studies (88%). The duration of training, when this was specified, ranged from 16–80 hours. Precise description of training was generally not provided; if mentioned, it was most often an intervention by a member of a professional network specialised in MI, such as the Motivational Interviewing Network of Trainers (MINT), for instance.^45^

Quality control (or fidelity) of the interviews was found in 17 out of 26 studies (65%). In the vast majority, specially trained facilitators used the Motivational Interviewing Treatment Integrity scale (MITI)^46,47^ to code the interviews, which had been previously recorded. To ensure a difference in intervention between the control and MI groups, two of the studies also included in their protocol the recording and coding of the sessions of the control groups to grade their ‘MI level’ and found a statistically significant difference between the groups.

#### Main outcomes

The main outcomes of the literature review are included in Table 3.

**Table 3.** Results in favour of motivational intervention according to outcome evaluation criteria

Criteria		Studies(*n* = 26)	Positive outcomes(*n* = 21, 81%)
Population	Children	8	6 (75%)
	Adolescents	18	15 (83%)
Presence of parents	Adolescents without their parents	6	5 (83%)
Outcomes	Clinical and anthropometric criteria	21	13 (62%)
	Behavioural criteria	17	12 (71%)
	Psychosocial criteria	7	5 (71%)
Location of the study	Studies in health centres	19	14 (74%)
	Non-medical-based interventions (school, church)	7	7 (100%)

Twenty-one out of 26 studies (81%) found a statistically significant result in favour of motivational intervention, all criteria considered.

Seventy-one per cent of the studies that included at least one behavioural criterion (dietary, physical, motivation, and so on) found a significant result in favour of the motivational intervention versus the control or intra-intervention group. This result is consistent with the main objective of MI, which is to generate behaviour change by creating motivation for change. This behavioural effect has the advantage of appearing quickly, being reported in studies of <6 months. These effects were found in children and adolescents, through modification of lifestyle and with cooperation of the parents. Several studies reported an improvement in ‘self-efficacy’, ‘feeling of personal efficacy’, and the feeling of support perceived by the patients. An improvement of these parameters is an essential step to maintain long-term adherence by the patient: by durably modifying the life habits, the weight effect will appear and be perpetuated.

Sixty-three per cent of the studies in this review found a beneficial effect of MI on anthropometric and/or clinical endpoints. As with behavioural effects, the results were observed in both children and adolescents, alone or with their parents. In contrast to lifestyle changes, anthropometric effects after MI require a longer time to be significantly measurable. The most frequently found indicator was the change in BMI in absolute value, percentile, or z-score. One-third of studies did not support weight improvement associated with behavioural changes, but study duration was often not long enough for this, with children or adolescents not receiving counselling for a prolonged period.

Seventy-one per cent of studies that examined psychosocial criteria reported a beneficial effect. One study clearly showed a decrease in depression scores in adolescents participating in MI, while control groups’ scores worsened.^48^ In the other studies, there was an improvement in quality of life or sense of self-efficacy.

Efficacy was globally associated with prolonged and regular follow-up of at least 6 months.

## Discussion

### Summary

The effectiveness of the MI advisory approach was reported in 81% of the studies. This statistically significant outcome is initially related to behavioural changes, and whether or not this approach is maintained, by anthropometric or biological effects.

These noteworthy results on MI in weight loss are consistent with those reported in previous literature on adults with obesity, wherein more than one-third of the studies had a statistically significant weight loss and about half had a 5% weight loss.^49^

Obesity is known to be a risk factor for social isolation and depressive disorders; at the same time, isolation and depression promote overweight.^49^ Having a tool that effectively addresses these disorders is an important lesson from this review and is consistent with the literature: stimulating intrinsic motivation appears to be an effective way to combat depressive symptoms.^50^

#### Modalities of the motivational approach

##### Regularity of the interviews

During repeated sessions, the effects measured were beneficial, but decreased with time without further consultation. This ‘dose-effect’ relationship seems to be proportional, as shown by two studies on parents and children aged 2–8 years and 3–5 years.^50,51^

##### Locations of care

All studies implemented in the school environment had noteworthy results and an excellent participation rate of >80%. This is an interesting perspective to fight against the attrition that often occurs with a follow-up in a healthcare centre and to achieve a higher frequency of consultation over a longer period. The value of school-based interventions has been found in the literature to reduce screen time, increase physical activity, and increase the ability to conduct a long-term follow-up.^52,53^

This involvement of the school environment is suggested in the recently updated recommendations of the Haute Autorité de santé (HAS; French National Authority for Health), for the early detection and management of overweight or obese young people.

##### Role of the parent

The findings of this review do not sufficiently elucidate the beneficial or detrimental effect of parental involvement in MI interventions when it comes to adolescent patients. These findings have led the authors to consider an alternative approach for adolescents: adolescents begin to feel a desire for independence and it is possible that the motivation to change is stronger in this population if it comes from them alone, because it is reinforced by a feeling of autonomy.^54^ The presence of parents may make them less comfortable in discussing intimate issues and is therefore questionable, as the effects are not necessarily beneficial.^55,56^

However, several studies of adolescents and parents in *Journal of Forensic and Legal Medicine* have shown the positive impact of maintaining parental support. Parental involvement seems to have a beneficial impact on behaviours, provided that the adolescent has received individual sessions (that is, without parental attendance).

##### Prerequisite skill to achieve effects

The higher the MI mindset scores of the practitioners, the higher the patients’ reported motivation to increase physical activity and improve diet. The present systematic review supports the importance of qualitative training to ensure that interventions include the maximum number of MI elements. Previously successful interventions were conducted by highly skilled practitioners. The challenge of technology transfer seems to be met in real-world conditions (and training in motivational interviewing for primary care physicians is achievable).^57^

### Implications for GP practice

#### General contributions of MI

Several studies have shown that the use of MI and mindful techniques correlate with better outcomes for patient behaviours.^58,59^ Other studies suggest MI training leads to less burnout and an increased sense of personal accomplishment for GPs.^60^

The results of this review encourage GPs towards an unrestricted use of MI to address obesity. This observation is also validated by the latest recommendations for good practice from HAS, which promotes support and motivation for change in a non-judgmental environment, which are the foundations of MI.^61^

#### Extended support

In addition to its early effects, MI promotes adherence and long-term follow-up.^62^ Its interest in chronic pathologies to fight attrition and demotivation is therefore essential. Several studies have proven its effectiveness in improving compliance with chronic conditions and are consistent with the results of this review.^63–65^

#### Full family involvement

The management of childhood obesity is a whole-family approach^65^ in which the family physician plays an important role. Children who are obese often have parents who are obese,^61^ and management of the child alone will have no impact if parental behaviours are not changed.

#### Multidisciplinary approach

The GP or the attending paediatrician has a central place in the follow-up of children and adolescents who are overweight. However, a multiprofessional approach is essential in order to ensure the feasibility of a regular long-term follow-up.^65^ As recommended by the HAS,^6^ integrating dieticians, physical therapists, adapted physical activity teachers trained in MI as well as doctors and nurses, will allow for a better treatment over several months or even years.

### Strengths and limitations

To the authors’ knowledge, this review of the literature is the first applied to management of obesity for both children and adolescents. A large number of articles have been included, allowing the exploration of numerous lessons regarding the results and the modalities of use of the MI. Most of the articles selected mentioned the training of their contributors and proceeded to a quality control of the MI via a coding of the sessions (MITI).

The articles using the MI, but not stating it in their title or abstract, were not included in the first screening (selection bias). The studies selected were heterogeneous (intervention, control, duration, evaluation criteria, and so on).

### Implications for practice

This review of the literature has shown that MI is a useful tool for the management of overweight and obesity in paediatric patients. Effects were found on behaviours and anthropometric values, the primary goal of the treatment.

It can be concluded that MI improves adherence to follow-up, has a high level of patient satisfaction with consultation experience, and leads to an improvement in psychosocial scores. In all cases, management must be family based and multiprofessional to achieve positive outcomes through a regular and varied follow-up. The inclusion of the school environment is promising because it facilitates access to care and improves compliance.

## Data

The data presented in this study are available on request from the corresponding author.
